# Treatment of Acute Myeloid Leukemia in the Community Setting

**DOI:** 10.1093/oncolo/oyae051

**Published:** 2024-08-19

**Authors:** Taha Al-Juhaishi, Servillano Dela Cruz, Rohan Gupta, Gina Keiffer, Vicki A Morrison, Iuliana Shapira, Ashley Woods, Kelly Norsworthy, Romeo Angelo de Claro, Marc R Theoret, Ravin Garg, Elizabeth Dianne Pulte

**Affiliations:** Oklahoma Health Sciences Center, University of Oklahoma, Oklahoma City, OK, USA; Florida Cancer Specialists & Research Institute, Inverness, FL, USA; The Center for Cancer and Blood Disorders, Fort Worth, TX, USA; Sidney Kimmel Cancer Center, Thomas Jefferson University, Philadelphia, PA, USA; Hennepin County Medical Center, University of Minnesota, Minneapolis, MN, USA; Regional Cancer Care Associates, Teaneck, NJ, USA; Center for Drug Evaluation and Research, U.S. Food and Drug Administration, Silver Spring, MD, USA; Center for Drug Evaluation and Research, U.S. Food and Drug Administration, Silver Spring, MD, USA; Center for Drug Evaluation and Research, U.S. Food and Drug Administration, Silver Spring, MD, USA; Oncology Center of Excellence, U.S. Food and Drug Administration, Silver Spring, MD, USA; Maryland Oncology and Hematology, Annapolis, MD, USA; Center for Drug Evaluation and Research, U.S. Food and Drug Administration, Silver Spring, MD, USA

**Keywords:** hematologic malignancy, leukemia, treatment options, community setting

## Abstract

The treatment landscape for acute myeloid leukemia (AML) is rapidly changing. Many new agents and lower-intensity regimens have been approved and can be safely used by hematologists and oncologists in both academic and community settings. The US Food and Drug Administration (FDA) held a virtual symposium on AML treatment in the community in November 2022. Several members of the FDA, along with practicing hematologists and oncologists in both academic and community settings, participated in the symposium. The goal of the symposium was to discuss challenges and opportunities in the treatment of patients with AML in community oncology settings. A summary of these discussions and key considerations are presented here.

## Teaser

The FDA held a virtual symposium on acute myeloid leukemia (AML) treatment in the community in November 2022 to discuss challenges and opportunities in the treatment of patients with AML in community oncology settings. A summary of these discussions and key considerations are presented here.

**Implications for Practice**

The US Food and Drug Administration (FDA) held a virtual symposium in November 2022 with a diverse group of hematologists and oncologists who practice in both academia and community settings. The symposium consisted of panel discussions between members of the FDA Division of Hematologic Malignancies I, which evaluates new therapeutic options for patients with acute and chronic leukemias, myelodysplastic syndrome, and hematopoietic stem cell transplantation, and community oncologists from institutions throughout the US. The panel discussed the current state and challenges in treating AML in the community setting in addition to future directions and considerations.

## Introduction

Acute myeloid leukemia (AML) is an aggressive hematologic malignancy that requires prompt identification and management. It primarily affects older patients with a median age of 69 at diagnosis.^1^ The outcomes of AML continue to improve somewhat over time with an overall 5-year expected survival of 32% in the last 10 years.^1^ The improvement in outcomes is attributed to several factors including development of effective and less toxic leukemia therapies including targeted agents, advancements in allogeneic hematopoietic stem cell transplantation (HSCT) that expanded eligibility and utilization, and progress in supportive care, especially infection control. Furthermore, recent data from a retrospective study suggested that delaying treatment initiation at the time of diagnosis to potentially allow results of genetics data to come back and guide therapy may not affect outcomes.^2^ Nevertheless, the outcomes of certain groups of patients with AML such as older or frail patients who are not candidates for intensive therapies or transplant, patients with therapy-related AML (AML with underlying myelodysplastic or myeloproliferative disease), multiply relapsed or refractory AML, or AML with TP53 mutation are still poor and only a minority of patients can obtain long-term disease control and survival.^3^

The field of leukemia has changed dramatically over the last 5 years with regulatory approval of many targeted agents and the ever-expanding field of drug development.^4^ There are a multitude of ongoing clinical trials for patients with AML testing many ideas from new combinations of old cytotoxic therapies to new targeted agents and immunotherapies, including T-cell engagers and chimeric antigen receptor (CAR)-T or NK-cell therapies. Furthermore, the field of HSCT, which remains the only curative therapy for high-risk or relapsed AML, has undergone multiple landmark milestones including the development of reduced-intensity conditioning regimens and the utilization of alternative donor transplants for patients without fully matched donors.^5^ This in turn has expanded transplant eligibility to older or less fit patients, and to patients of ethnic minorities who usually have difficulties finding suitable donors in donor registries.^6^ The changes in AML treatment and supportive care options have improved outcomes for patients. However, the proliferation of new options, some with unique risks, may create confusion in terms of which treatment options are optimal in various circumstances. Historically speaking, the treatment of AML was primarily taking place at large academic centers that have resources to deliver more toxic therapies such as intensive induction and HSCT. Data on the treatment of AML in the community setting remain limited; however, the development of new lower-intensity options has allowed community oncologists to participate more actively in the treatment of patients with AML.^7^

To address the current practices and challenges of AML treatment in the community setting, the Food and Drug Administration (FDA) held a virtual symposium in November 2022 with a diverse group of hematologists and oncologists who practice in both academia and community settings. The symposium consisted of panel discussions between members of the FDA Division of Hematologic Malignancies I, which evaluates new therapeutic options for patients with acute and chronic leukemias, myelodysplastic syndrome, and HSCT, and community oncologists from institutions throughout the US. The panels focused on AML treatment in the community setting, clinical trial access in the community setting, and the FDA’s role in the care for patients with AML. We present here a review of the literature and highlights of the meeting including future directions and considerations.

## Current Practices and Challenges

AML is considered a rare malignancy constituting 1% of all new cancer cases with an expected incidence of 20 000 cases in the US in 2023.^1^ The complexity and clinical aggressiveness at initial presentation for many patients, in addition to low incidence of the disease, have historically contributed to limiting the treatment of AML to centers dedicated to leukemia care and research. Furthermore, the practice of HSCT is usually confined to large leukemia centers and major academic institutions, again prompting community hematologists and oncologists to refer most patients to an academic center once a diagnosis of AML is suspected. However, once the diagnosis is established, it is prudent to determine whether the patient is a candidate for intensive therapies including HSCT based on age, comorbidities, adequate organ function, and performance status. For patients who are candidates for HSCT, the process for donor search should be started as early as possible to account for potential future delays in donor evaluation and stem cell collection.

It is important to highlight that there are no universally accepted criteria to accurately determine who would be or not be a candidate for intensive therapies, an issue that has been long recognized in the context of AML clinical trials. However, several models have been developed to help in clinical decision-making.^8,9^ Generally speaking, if the patient is judged to be a candidate for intensive therapies, then they should be referred to a leukemia and transplant center for discussions of treatment strategies including the role of HSCT, and to start the process of donor search which could be lengthy for many patients.

**Figure 1.**
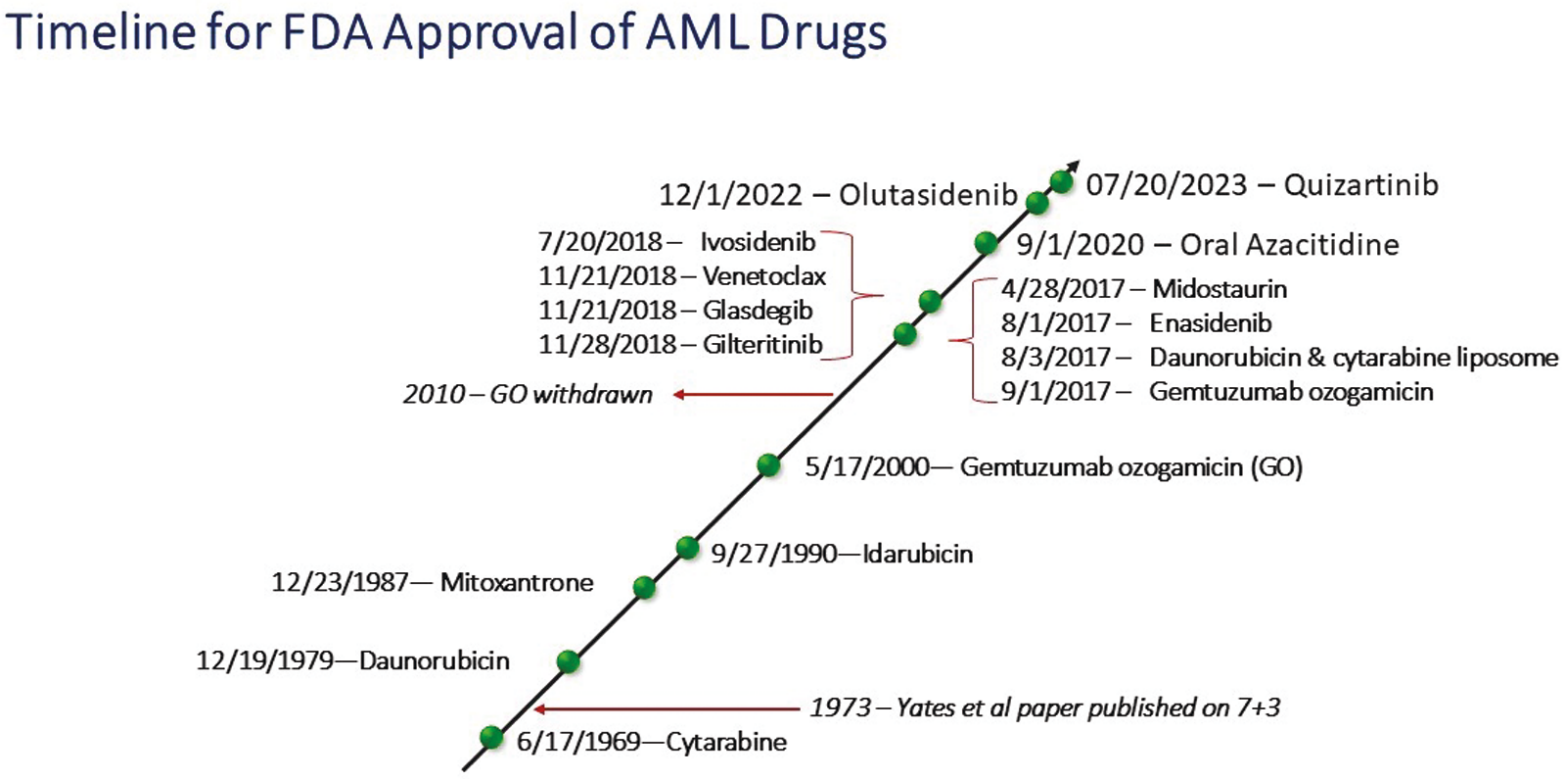
FDA-approved drugs in AML^10-20^

The role of clinical trials is also essential, especially knowing that many patients with AML will still succumb to their disease despite all the progress made in understanding and treating this disease. Many of these trials are, unfortunately, only available at large leukemia centers. Of note, the development of less toxic drugs and lower-intensity treatment strategies, especially in the last 5 years, has allowed many community hematologists and oncologists to successfully treat patients with AML. This is especially true for patients who are older or unfit, who may have a hard time relocating to receive care at a large leukemia center, and who may not be eligible or interested in enrolling in clinical trials. Specifically, the combination of a hypomethylating agent (azacitidine or decitabine) or low-dose cytarabine (LDAC) combined with the BCL2 inhibitor, venetoclax, LDAC combined with the hedgehog inhibitor, glasdegib, and molecularly targeted agents such as the IDH1 inhibitor ivosidenib, as single agent or in combination with azacitidine, are increasingly popular regimens that can be well suited for community practice. These regimens are designed for outpatient administration and can have lower toxicities, including infections and mucositis when compared to intensive induction chemotherapy regimens. These regimens have also been shown to be quite effective and safe in patients planned to undergo HSCT. Examples of approved targeted agents include the IDH1 inhibitors ivosidenib and olutasidenib, the IDH2 inhibitor enasidenib, and the FLT3 inhibitor gilteritinib^10-19^ (Fig. 1).

Logistical difficulties are also commonly seen in community care of patients with AML. Insurance coverage, including timely preauthorization approvals, can be quite challenging regarding some of the high-cost diagnostic testing and therapies. Specifically, the use of comprehensive gene panels to look for targetable mutations and the use of targeted agents in a timely manner can be difficult for community hematologists and oncologists due to many reasons including financial coverage. Some potential solutions to these issues include the development of shared care models wherein patients with AML can be comanaged by a leukemia/transplant specialist and their community hematologist and oncologist to make sure patients have access to all available therapeutic opportunities in a manner that aligns with their wishes and can protect their quality of life.

## Management of Treatment Toxicities

It is important to point out some of the common toxicities seen with these recently approved regimens that community hematologists and oncologists need to become familiar with both in terms of recognition and management: tumor lysis syndrome and prolonged myelosuppression, especially with BCL2 inhibitors, and differentiation syndrome and prolonged QTc interval, especially with IDH and FLT3 inhibitors.^10-16,19^ The importance of ensuring that community providers who treat AML understand these risks was discussed. In particular, differentiation syndrome, which was previously observed only in patients with acute promyelocytic leukemia (APL) being treated with all *trans*-retinoic acid (ATRA), can be seen with molecularly targeted agents including ivosidenib, enasidenib, and gilteritinib.^21^ This syndrome may pose challenges to the community oncologist for several reasons. First, because community oncologists treat a variety of malignancies and only rarely treat AML, they may have difficulty promptly recognizing and treating it. Second, differentiation syndrome related to ATRA is typically seen at a predictable time early in the course of treatment of APL, whereas targeted therapy-induced differentiation syndrome may occur at almost any time from within a few days of the start of treatment to months into treatment.^6^ Finally, because many of the symptoms of differentiation syndrome overlap with symptoms of infection or fluid overload, there is a risk of misdiagnosis, especially if the patient presents at an outside emergency room where the treating physician may not know the patient’s medical history or the implications of treatment with molecularly targeted agents.

Supportive care, including transfusion support and infection prevention and control, especially against fungal and opportunistic infections, is extremely vital in leukemia management and can be more challenging in the community setting. It is important to highlight that myelosuppression is still a significant toxicity even with newer less-intensity regimens that combine hypomethylating agents with venetoclax. Patients may require multiple red cell transfusions and/or platelet transfusions per week. Given that, patients may need to present to the clinic twice per transfusion, once for type and cross, and once for the actual transfusion which is a considerable amount of time spent in the clinic. Furthermore, finding compatible units may be difficult in patients who have alloimmunization. Similarly, treatment of infection may be more difficult in the community setting where access to infectious disease specialists and other support may be less than in the academic setting Notably, drug interactions like with antifungals and novel agents such as venetoclax require appropriate dose adjustments to avoid toxicities. Finally, the COVID19 pandemic added difficulties in the management of AML, both in terms of further limiting resources including intensive care unit space, and adding another infectious risk. Thus, providing patients with vaccinations and being up to date on best practices for the prevention and treatment of COVID19 infection is crucial.

## Clinical Trials Access in the Community Setting

Clinical trials are an essential part of the management of patients with AML especially those with higher-risk disease and worse prognosis. Historically, developing new drugs in AML was a daunting task and the field was static for decades. However, 11 new approvals were granted between 2017 and 2023. There are currently many new drugs in different stages of development and the number of active trials is rising fast. There are 2 main areas in drug development for AML that warrant highlighting:

1. Genomics-based approaches or what is referred to as “precision medicine” have resulted in multiple approvals already, including several small-molecule inhibitors of specific mutations.
2. Immuno-oncology; despite the recognition of the curative power of the immune system in patients with AML—as seen in graft-versus-leukemia (GVL) effect post HSCT—the development of immunotherapy-based drugs in AML has been challenging. Multiple studies evaluated the role of immune checkpoint inhibitors in AML but unfortunately no clear benefit was seen.^22^ Recently, the development of cellular immunotherapies such as T-cell engagers or CAR T-cell therapies have shown to be effective in B-cell and plasma cell malignancies; however, their development in myeloid disease is still in early phases.^23,24^

Access to clinical trials in the community faces multiple challenges. Most clinical trials are only open and exclusively run at major leukemia centers and so patients need to be referred and must relocate or travel frequently. Furthermore, the eligibility and exclusion criteria for many trials can be challenging. Most patients with AML are above the age of 65 and can have many chronic conditions especially heart, lung, and kidney disease that might make them ineligible for many studies. Other strict requirements such as multiple repeated marrow biopsies and delays in initiation of therapies until additional tumor genetic testing is completed which pose additional barriers to accrual, especially in patients with symptomatic or rapidly progressive disease. Furthermore, delays in therapy due to the need for procedures or confirmation of specific genetic markers could result in patients with less aggressive disease selectively enrolling in clinical trials, compromising the external validity of the results. It is important to simplify the eligibility and exclusion criteria to improve accrual and add more external validity to the results of clinical trials.

Decentralizing the conduct of clinical trials in the future, especially for rare and morbid diseases like AML should be emphasized. This would particularly encourage enrollment of older or less fit patients in clinical trials given that they may otherwise have difficulty relocating to participate. Good candidates for these decentralized trials include studies of drugs with a favorable safety profile and relative ease of administration Of note, the FDA recently released a guidance document on the implementation of decentralized clinical trials.^25^ The role of payers is also vital including making sure that different payers are committed to providing coverage for any requirements that are otherwise not covered by studies as this should never be the responsibility of the patients. Lastly, these studies should be designed and statistically powered to answer a clinical question that matters to the patients such as improvement in overall survival or quality of life, and the use of earlier clinical benefit endpoints such as event-free survival or response endpoints should be minimized.

## Future Directions

The field of AML is growing fast, and therapy is no longer limited to conventional cytotoxic chemotherapies and HSCT for patients who are fortunate to have a responsive disease and a suitable donor. This positive momentum can only be sustained through contributions by different stakeholders including patients, treating physicians both in academia and community health care systems, regulatory agencies, payers, and the pharmaceutical industry.

AML is no longer a disease that can only be treated at large academic leukemia centers and the role of community care will continue to grow. It is crucial for all stakeholders to work together to overcome barriers to patients accessing the best available diagnostics, therapeutics, and clinical trials. Constant collaboration between academia and community care is necessary to eliminate disparities. Sufficient and ongoing education efforts to narrow the gap between the specialized expertise in academic centers and their colleagues in the community are highly needed. Furthermore, it is highly encouraged to involve community hematologists and oncologists in the designs of future clinical trials in AML as they can provide critical input on potential challenges to wider utilization of therapies of interest in the community setting. It is also important to continue to study outcomes of patients with AML treated in both academia and community settings and use real-world data to guide decision-making for future areas of improvement. Finally, the financial toxicities of leukemia therapies can be significant, and different stakeholders should continue to work on developing paradigms for more cost-effective care (Table 1).

**Table 1.** Key considerations for the treatment of AML in the community.

1. Most targeted agents and lower-intensity chemotherapy regimens are well-suited for outpatient administration and can be used in different community settings
2. Providing appropriate supportive care such as frequent blood transfusions and aggressive management of infections is crucial for long-term success in patients with AML and can be done in collaboration with larger nearby leukemia centers
3. Close collaboration with local HSCT centers is encouraged in order to provide input on eligibility for HSCT and comprehensive care for patients who undergo HSCT as part of their treatment
4. Participation in clinical trials is highly encouraged
5. Clinical trials in AML should evolve to include decentralization of conduct and more inclusive and flexible eligibility requirements

## Summary

Treatment options for AML will continue to expand especially in terms of molecular targeted agents and cellular immunotherapies. It is no longer viable to limit the treatment of AML to large academic leukemia centers and the role of community care will continue to grow. Clinical trials should also evolve and expand both accrual and the conduct of studies to community clinics with close collaboration with nearby large academic and research institutions. All different stakeholders are required to actively participate in the path to cure AML for all patients.

## Data Availability

The data underlying this article are available in the article.
